# *CircZNF609* and *circNFIX* as possible regulators of glioblastoma pathogenesis via *miR-145-5p*/*EGFR* axis

**DOI:** 10.1038/s41598-024-63827-w

**Published:** 2024-06-12

**Authors:** Elham Ghadami, Ali Gorji, Ahmad Pour-Rashidi, Farshid Noorbakhsh, Majid Kabuli, Masoumeh Razipour, Hamid Choobineh, Mohaddese Maghsudlu, Elia Damavandi, Mohsen Ghadami

**Affiliations:** 1 Department of Medical Genetics, School of Medicine, Tehran University of Medical Sciences, Tehran, Iran; 2 Shefa Neuroscience Research Center, Khatam Al-Anbia Hospital, Tehran, Iran; 3 Department of Neurosurgery, Westfälische Wilhelms-Universität, Munster, Germany; 4 Department of Neurosurgery, Sina Hospital, Tehran University of Medical Sciences, Tehran, Iran; 5 Department of Immunology, School of Medicine, Tehran University of Medical Sciences, Tehran, Iran; 6 School of Allied Medicine, Tehran University of Medical Sciences, Tehran, Iran; 7 Department of Photodynamic, Medical Laser Research Center, Yara Institute, ACECR, Tehran, Iran; 8 Specialized Medical Genetic Center (SMGC) of ACECR, Tehran, Iran; 9 Cardiac Primary Research Center, Tehran Heart Center, Tehran University of Medical Sciences, Tehran, Iran; 10 Endocrinology and Metabolism Research Institute, Tehran University of Medical Sciences, Tehran, Iran

**Keywords:** Glioblastoma, *CircZNF609*, *CircNFIX*, *miR-145-5p*, *EGFR*, Cancer, Genetics

## Abstract

Glioblastoma is a rare and deadly malignancy with a low survival rate. Emerging evidence has shown that aberrantly expressed circular RNAs (circRNAs) play a critical role in the initiation and progression of GBM tumorigenesis. The oncogenic function of *circZNF609* and *circNFIX* is involved in several types of cancer, but the role and underlying mechanism of these circRNAs in glioblastoma remain unclear. In this study, we hypothesized that *circZNF609* and *circNFIX* may regulate *EGFR* through sponging *miR-145-5p*. Herein, we assessed the expression levels of *circZNF609*, *circNFIX, miR-145-5p*, and *EGFR* using quantitative polymerase chain reaction in glioblastoma patients and normal brain samples. The results showed that *circZNF609*, *circNFIX,* and *EGFR* expression levels were upregulated and *miR145-5p* was downregulated (p = 0.001, 0.06, 0.002, and 0.0065, respectively), while there was no significant association between clinicopathological features of the patients and the level of these genes expression. We also found a significant inverse correlation between *miR145-5p* and the expression of *cZNF609*, *cNFIX* and *EGFR* (p = 0.0003, 0.0006, and 0.009, respectively). These findings may open a new window for researchers to better understand the potential pathways involved in GBM pathogenesis. In conclusion, it may provide a new potential pathway for the development of effective drugs for the treatment of GBM patients.

## Introduction

Glioblastoma (GBM) is a grade IV glioma according to the 2021 WHO classification and is the most aggressive and lethal primary malignancy of the central nervous system^1^. Despite recent advances in exploring novel therapeutic approaches for GBM, the outcomes of most patients are unfavorable^2^. The median survival from initial diagnosis is less than 15 months with treatment. Thus, further understanding of the underlying molecular mechanisms is needed to help develop effective therapeutic strategies for the treatment of GBM^3^.

Recently, a growing number of studies have shown that circular RNA (circRNA) plays an important regulatory role in developing and progressing cancer, including glioblastoma^4^. CircRNA is an endogenous, highly stable, single-stranded closed circular RNA molecule without either a 5′ cap or 3′ polyadenylate tail. CircRNAs are divided into four major types: exonic circRNAs (ecRNAs), circular intronic RNAs (ciRNAs), exon–intron circRNAs (EIciRNAs), and intergenic circRNAs, according to the sequence origin^4,5^.

CircRNAs mainly act as miRNA sponges to modulate downstream mRNA through the constructed a circRNA-miRNA-mRNA (ceRNA) network^5^. Among the cancer-related circRNAs, *circZNF609* and* circNfIX* functioned as onco-circRNAs, the upregulation of which facilitated cancer cell proliferation and migration by regulating miRNAs*.*^6,7^. MicroRNAs (miRNAs), a subset of non-coding RNAs conserved across species, regulate gene expression by binding to the 3′-untranslated region of target mRNAs^8^. In recent years, many studies focused on the regulatory role of miRNAs as the core of the competitive endogenous RNA (ceRNA) hypothesis that explains the relationship between different classes of RNAs including circRNA, long noncoding RNA (lncRNA), miRNAs and mRNAs^9^. *Hsa-miR-145-5p*, one of the targets of several dysregulated circRNAs identified in studies, has been widely confirmed to be associated with cancer^8,10^. Evidence has shown that *miR-145-5p* can be regulated by different mechanisms and can involve in tumorigenesis and progression through binding to 3′-UTRs of target mRNAs^10,11^. Several studies have reported that *miR-145-5p* can significantly suppress tumor progression, including in colorectal cancer (CRC)^12^, lung cancer^13^, and glioma^14^ via targeting epidermal growth factor receptor (EGFR) gene. EGFR is a trans-membrane receptor tyrosine kinase of the ERBB family and has a role in cell proliferation, differentiation and cell survival. An overexpression of either *EGFR* or *EGFR*-activating mutations is associated with poor clinical prognosis in various human tumors^15,16^. Despite many reports on the molecular mechanism of EGFR, circRNA-induced EGFR regulation in glioblastoma is still poorly understood.

In the current study, using literature review and bioinformatics analysis from different web tools and databases including NCBI Gene Expression Omnibus (GEO) (https://www.ncbi.nlm.nih.gov/geo/), Circular RNA Interactome database (https://circinteractome.irp.nia.nih.gov/), miRNet (http://www.mirnet.ca) and IntaRNA web servers (https://rna.informatik.uni-freiburg.de/IntaRNA/Input.jsp), and miRTarBase (http://miRTarBase.cuhk.edu.cn/), we predicted the presence of a regulatory axis, namely, *circZNF609*, *circNFIX*/*miR-145-5p*/*EGFR* axis. *CircZNF609* was the alias of *hsa_circ_0000615* which derived from the host gene *ZNF609*. Dysregulation of *CircZNF609* has been reported in a few cancers such as lung adenocarcinoma^17^, nasopharyngeal carcinoma^18^, and hepatocellular carcinoma^19^. Another circRNA, *hsa_circ_0049658*, originated from host gene nuclear factor IX (*NFIX*) mRNA. Previous studies have shown that *circNFIX* is upregulated in liver cancer, non-small cell lung cancer and glioma, and functions as an oncogene involved in cancer metabolism, chemotherapy resistance, migration and invasion^20^. However, the influence and mechanism of these circRNAs in glioblastoma needs further research, and whether *circZNF609* and *circNFIX* may play a role as ceRNAs in GBM pathogenesis remains to be investigated. So, we hypothesized that *circZNF609* (*circ0000615)* and *circNFIX* (*circ0049658*) regulate *EGFR* expression through sponging *miR-145-5p*. The relative expressions of *circZNF609*, *circNFIX*, *miR-145-5p* and *EGFR* were then evaluated in glioblastoma compared to normal brain tissues. Also, the relationship between the expression of these genes and the clinical features of patients such as age, gender and tumor diameter was assessed.

## Materials and methods

### Specimens collection and ethics

A total of 51 samples, including 30 tumor tissues from glioblastoma patients and 21 normal brain tissues were collected from the Department of Neurosurgery of Sina Hospital and Shafa Neuroscience Research Center at Khatam Al-Anbia Hospital. All tissue samples were diagnosed by two independent pathologists. All procedures performed in studies involving human participants were in accordance with the ethical standards of the institutional and/or national research committee and with the 1964 Helsinki declaration and its later amendments or comparable ethical standards. The study was approved by the Ethical Committee of Tehran University of Medical Sciences (Ethical code: IR.TUMS.MEDICINE.REC.1401.487), and written informed consent was obtained from all subjects. None of the patients underwent radiotherapy or chemotherapy before surgery. The clinicopathological features including age, gender, and tumor size of patients were attained from their medical records. Control brain samples were taken from patients with a kind of cerebral hemorrhage that have needed surgery and cerebral lobectomy at the same time. The characteristics of the study population are shown in Table 1.

**Table 1:** Clinicopathological features of cases.

Characteristic	Values	Tumoral	Normal
Gender	Male	25 (83.33%)	11 (52.38%)
	Female	5 (16.67%)	10 (47.62%)
Age	≤ 49	12 (40%)	13 (61.91%)
	> 49	18 (60%)	8 (38.09%)
Tumor size, cm	< 5	17 (56.67%)	–
	≥ 5	13 (43.33%)	
Sum		30	21

### Bioinformatics analysis and primer design

We screened differential miRNAs in glioblastoma and normal tissues from the GEO dataset (GSE165937, NCBI) using the edgeR package in R software with |logFC|≥ 1, adjust P value < 0.05. Volcano diagram of differentially expressed miRNAs was plotted using ggplot2.

To investigate the relationships between miR-145-5p and its target genes, we used MiRNet (http://www.mirnet.ca), a user-friendly and powerful tool that supports different types of miRNA targets for network creation and analysis.

The interaction between *cZNF609* (*circ0000615) and cNFIX (circ0049658) with miR-145-5p* was performed with online software including Circular RNA Interactome database (CRI, https://circinteractome.irp.nia.nih.gov/) and IntaRNA (https://rna.informatik.uni-freiburg.de/IntaRNA/Input.jsp)^21^. In addition, miRanda (https://regendbase.org/tools/miranda) and miRTarBase (http://miRTarBase.mbc.nctu.edu.tw/) were used to predict the downstream target of *miR-145-5p*. According to the complementary regions between the *circ0000615*, *circ0049658*, *miR-145-5p* and *EGFR*, we hypothesized that these circRNAs regulates *EGFR* by sponging *miR-145-5p* and thus, we considered the *cZNF609*, *cNFIX*/*miRNA-145-5p*/*EGFR* axis for further experiments.

The suitable primers were designed with Primer Blast and Gene Runner software. Moreover, the specificity of divergent primers for *hsa_circ_0000615* and *hsa_circ_0049658* was checked by circPrimer 1.2 software. The sequences of primers in this study are shown in Table 2.

**Table 2:** Sequences of primers.

Target transcript	Primer type	Sequences (5′-3′)
*circZNF609*	Forward	TTGGGAACTAAACCGGAGCC
*circZNF609*	Divergent reverse	TCAGACCTGCCACATTGGTC
*circNFIX*	Forward	TTGTGCAGTTTGTGTGCTCG
*circNFIX*	Divergent reverse	GAAACTTAAGTGCCCGTTGGG
*miR-145-5p*	Stem-loop RT	GTCGTATCCAGTGCAGGGTCCGAGGTATTCGCACTGGATACGACAGGGAT
*miR-145-5p*	Forward	GGCTTAGTCCAGTTTTCCCAG
*miR-145-5p*	Reverse	GTGCAGGGTCCGAGGT
*U6*	Stem loop RT	AACGCTTCACGAATTTGCGT
*U6*	Forward	CTCGCTTCGGCAGCACA
*U6*	Reverse	AACGCTTCACGAATTTGCGT
*EGFR*	Forward	ACAGCTATGAGATGGAGGAA
*EGFR*	Reverse	CACCAATACCTATTCCGTTACA
*GAPDH*	Forward	AAGGCTGAGAACGGGAAGCT
*GAPDH*	Reverse	CAGCATCGCCCCACTTGATT

### RNA extraction and quantitative real-time PCR

Approximately 15 mg of frozen tissue samples using liquid nitrogen, were homogenized. Total RNA was then extracted using Kiazol reagent (Kiazist, Iran) according to the manufacturer's instructions, and then quantity and purity of RNAs were measured by Nanodrop 2000C (Thermo Scientific, USA) and electrophoresis on 1.8% (wt/vol) agarose gel. RNA samples were converted into cDNA using the cDNA synthesis kit (Yekta Tajhiz Azma, Iran). Reverse transcription of mRNA and miRNA was performed using random primers and specific stem–loop primers, respectively. The reaction condition was set at 70 °C for 5 min, 42 °C for 60 min, and 70 °C for 5 min.

The relative expression levels of all genes were evaluated using RealQ Plus 2 × Master Mix Green without Rox (Ampliqon, Denmark) using the LightCycler 96 (Roche, Germany). Thermal cycling conditions for *circZNF609*, *circNFIX* and *EGFR* include preincubation at 95 °C for 900 s followed by amplification in 40 cycles at 95 °C for 15 s and 60C for 50 s. The suitable annealing temperature for *miR-145-5p* was 58 °C. *SnRNA U6* as a housekeeping gene for *miR-145-5p*; and *GAPDH* as a reference gene for circRNAs and *EGFR*. PCR products were analyzed by melting curve analysis and agarose gel electrophoresis. The back-splice junction sequences of *circZNF609* and *circNFIX* were confirmed by Sanger sequencing. All of the qPCR reactions were performed in duplicate and data were calculated by comparing Ct values.

### Statistical analysis

All statistical analysis was performed using GraphPad Prism 8.0 (GraphPad Software, Inc., San Diego, CA) software. Normality was checked by the Shapiro–Wilk test. The relative expression of *circZNF609*, *circNFIX*, *miR-145-5p*, and *EGFR* was evaluated in glioblastoma tumors and normal brain tissues using the unpaired sample t test. The association between the expression of circRNAs, *miR-145-5p* and *EGFR* with clinicopathological features of patients was calculated using Mann–Whitney and one-way analysis of variance (ANOVA) tests. Pearson correlation coefficient was used for the correlation of expression of genes analyses. Also, the receiver operating characteristic (ROC) curve was employed to investigate the diagnostic potential of circRNA biomarker(s) by the GraphPad Prism v.8 software. A value of p < 0.05 was considered statistically significant.

## Results

### Clinicopathological features

The median age of participants was 49 years (range, 12–75 years). The gender ratio of male to female patients was 5:1. The average ages at the time of diagnosis were 51.88 ± 15.87 and 55.8 ± 12.25 years for male and female patients, respectively. Most tumors were found in the frontal and temporal lobes of the brain, in two cases the tumor was located in the brain stem, which were diagnosed at a young age. No family history of brain cancer or other types of cancer has been found in patients.

### MiR-145-5p expression was decreased in glioblastoma tissues

**Figure 1:**
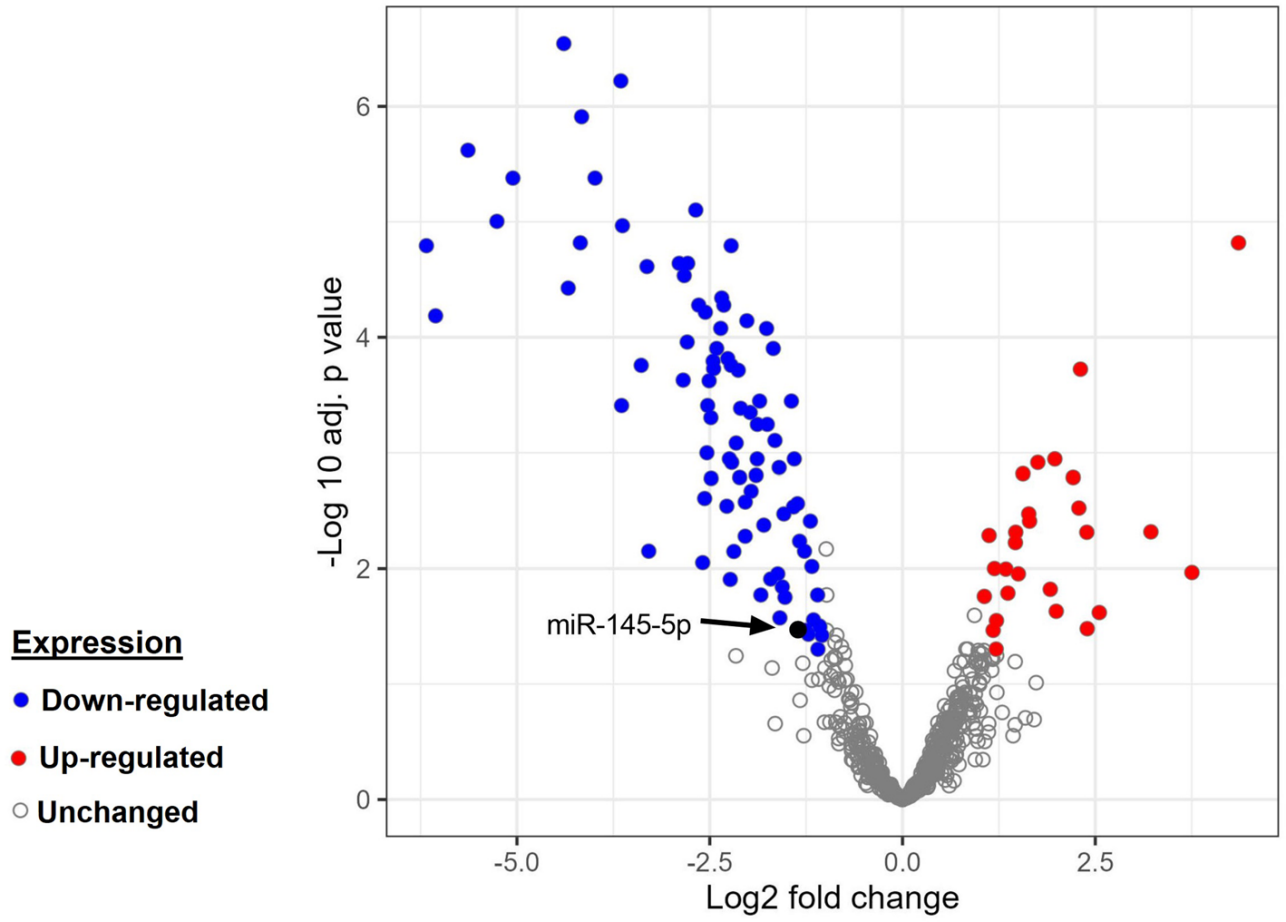
Volcano diagram of differentially expressed miRNAs in glioblastoma. |log2FC|> 1 and adj p-value < 0.05 were considered as cut-off values for the identification of DE-miRNAs. Blue and red dots denote the downregulated and upregulated miRNAs in glioblastoma samples; grey dots denote miRNAs without differential expression between GBM and normal samples. The arrow represents the miR-145-5p.

Using bioinformatic analysis of raw data from the GEO dataset, we investigated the expression levels of miRNAs in GBM tissues compared to normal brain tissues (Supplementary Table 1). According to hypothesis of this study, we focused on *miR-145* analysis. The results indicated that the expression of *miR-145-5p* was significantly lower in glioblastoma tissues than in normal tissues (log FC: − 1.25, adj p value = 0.0342) (Fig. 1).

### miRNA–target interactions

**Figure 2:**
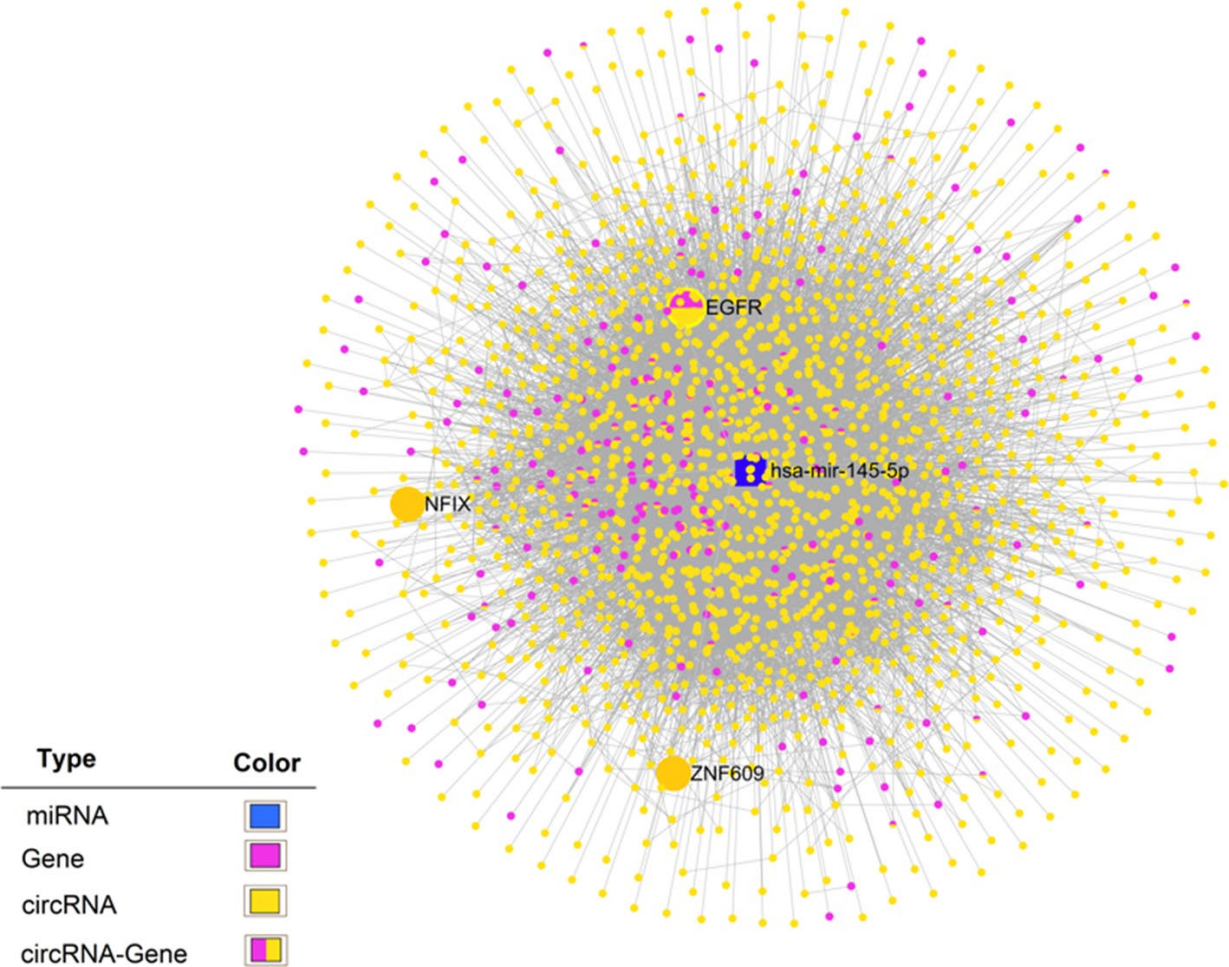
Predicted target genes of *miR-145-5p*. miRNet was used for establishing a network of *miR-145-5p* and their target genes. Blue rectangle represents *has-mir-145-5p*; purple and orange dots represent target genes and circRNAs; the dots shown with two colors represent circRNA-gene.

**Figure 3:**
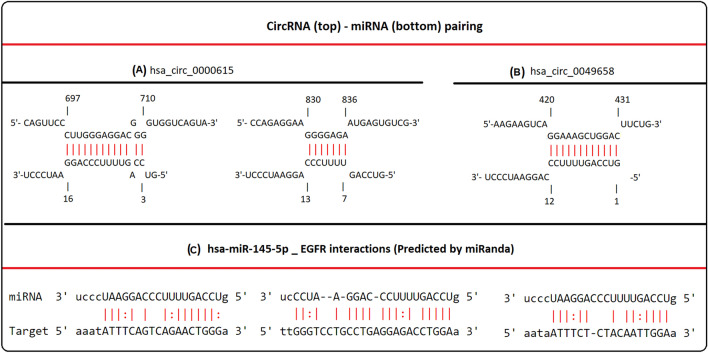
*Circ-ZNF609* and *circ-NFIX* may act as ceRNAs to bind to *miR-145-5p* with *EGFR*. Interaction sites between (**A**) *circ0000615*, (**B**) *circ0049658* and *miR-145-5p* according to IntaRNA. (**C**) Three interaction sites between *miR-145-5p* and *EGFR* based on bioinformatics website (miRanda and miRTarBase).

The interaction between *hsa-miR-145-5p* and its target genes, which was performed using miRNet, represented as a network. The resulting miRNA-target interactions showed that 1602 circRNAs and 238 genes interact with *hsa-miR-145-5p*. The genes investigated in this study were indicated as bigger nodes (Fig. 2). We hypothesized that *circZNF609* and *circNFIX* regulates EGFR through sponging *miR-145-5p*. The interaction sites between *circZNF609* (*circ0000615*) and *circNFIX* (*circ0049658*) with *miR-145-5p* and also binding sites between *miR-145-5p* and *EGFR* are illustrated in Fig. 3A–C), respectively.

### Evaluating the expression differences of *cZNF609*, *cNFIX*, *miR‑145* and *EGFR* between glioblastomas and normal tissues

**Figure 4:**
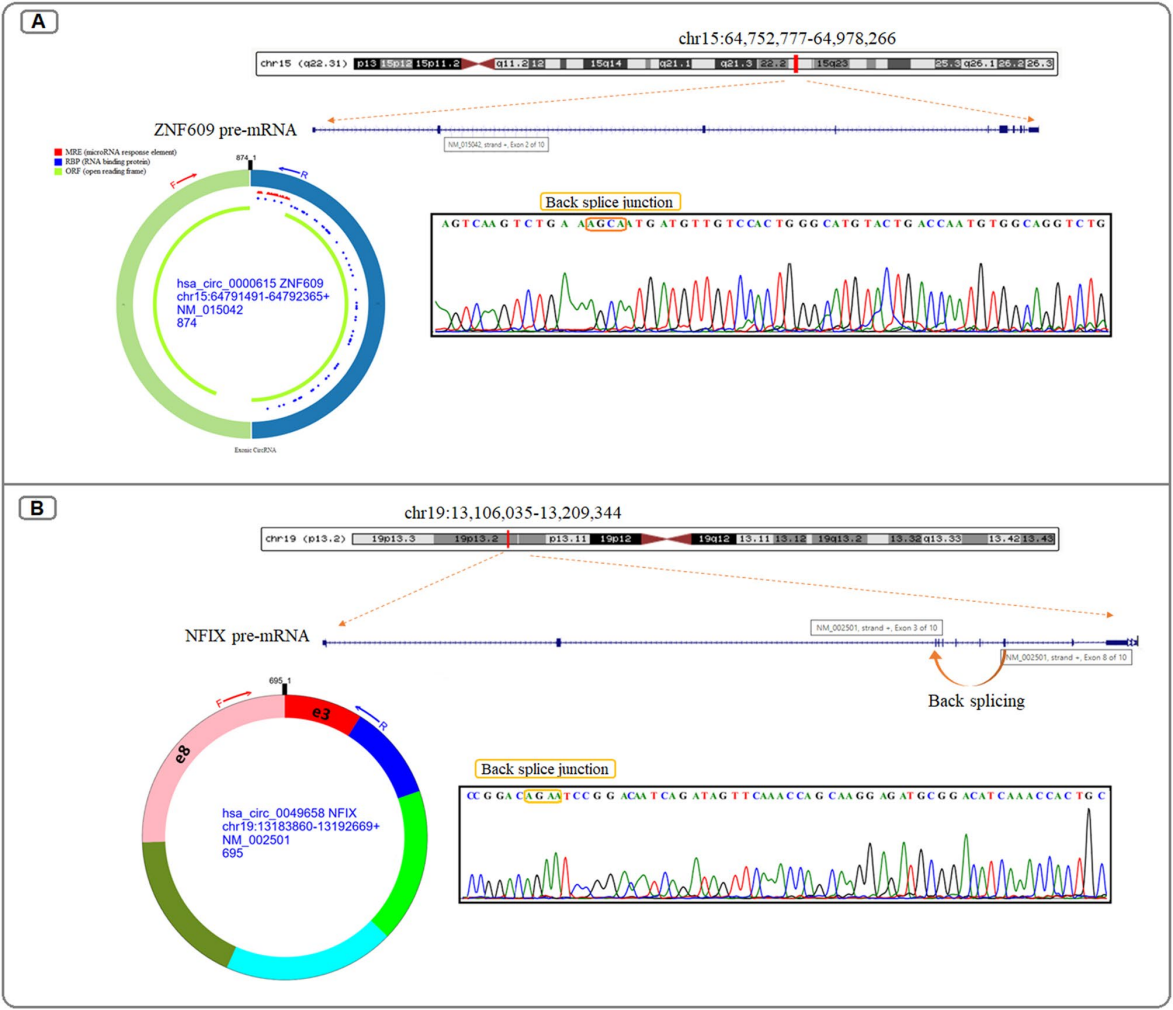
Schematic representation of (**A**) *circ0000615* and (**B**) *circ0049658* biogenesis. The back splice junction site of these circRNAs was confirmed by Sanger sequencing.

**Figure 5:**
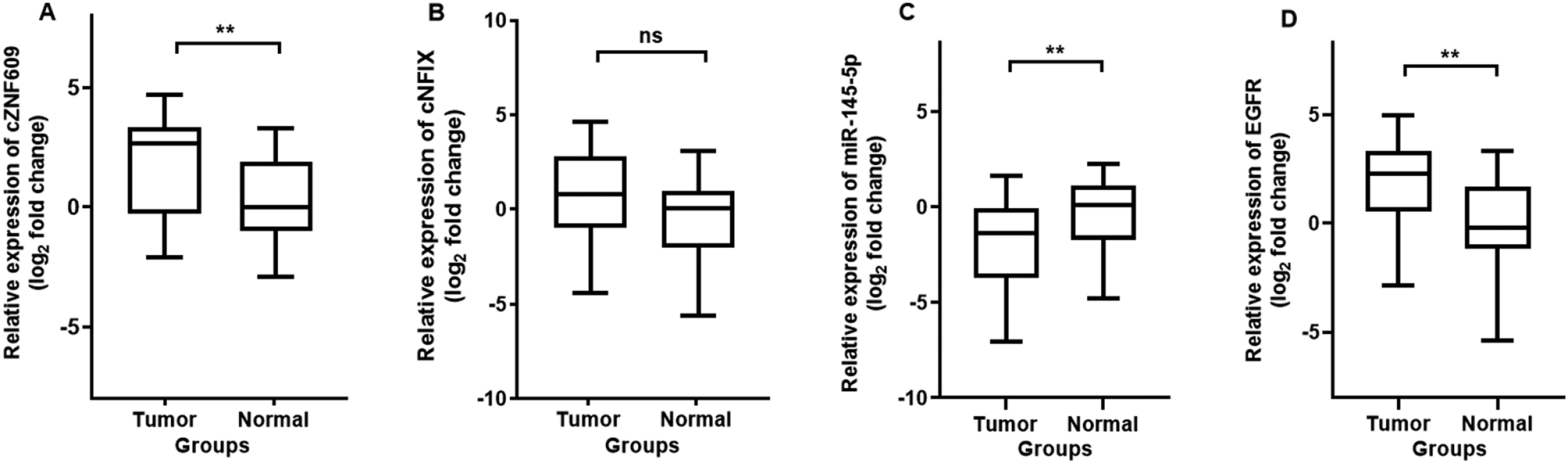
Relative expression of (**A**) *circZNF609*, (**B**) *circNFIX*, (**C**) *miR-145-5p* and (d) *EGFR* genes in glioblastoma samples, compared with normal brain tissues. ^**^ indicates a significant difference between two groups (p < 0.01) and ns indicates that not statistically significantly different among the groups (p > 0.05).

*CircZNF609* (*hsa_circ_0000615*) is derived from the circulation of the second exon of *ZNF609*. This back splice junction site was verified by the Sanger sequencing (Fig. 4A). *CircNFIX* (*hsa_circ_0049658*) is reverse spliced from the mRNA exons 3-8 of the host gene nuclear factor IX (*NFIX*) (Fig. 4B). Expression analysis showed a significant upregulation of *circZNF609* in GBM samples compared to normal brain tissue (p = 0.001) (Fig. 5A), whereas *circNFIX* gene expression was increased in tumor samples compared to normal samples, but not significantly (p = 0.06) (Fig. 5B). We also found downregulation of *miR-145-5p* (p = 0.0065) (Fig. 5C) and upregulation of *EGFR* (p = 0.002) in GBM samples compared to normal tissue (Fig. 5D).

### Correlation analysis between expression levels of *cZNF609*, *cNFIX*, *miR-145-5p*, and *EGFR*

**Figure 6:**
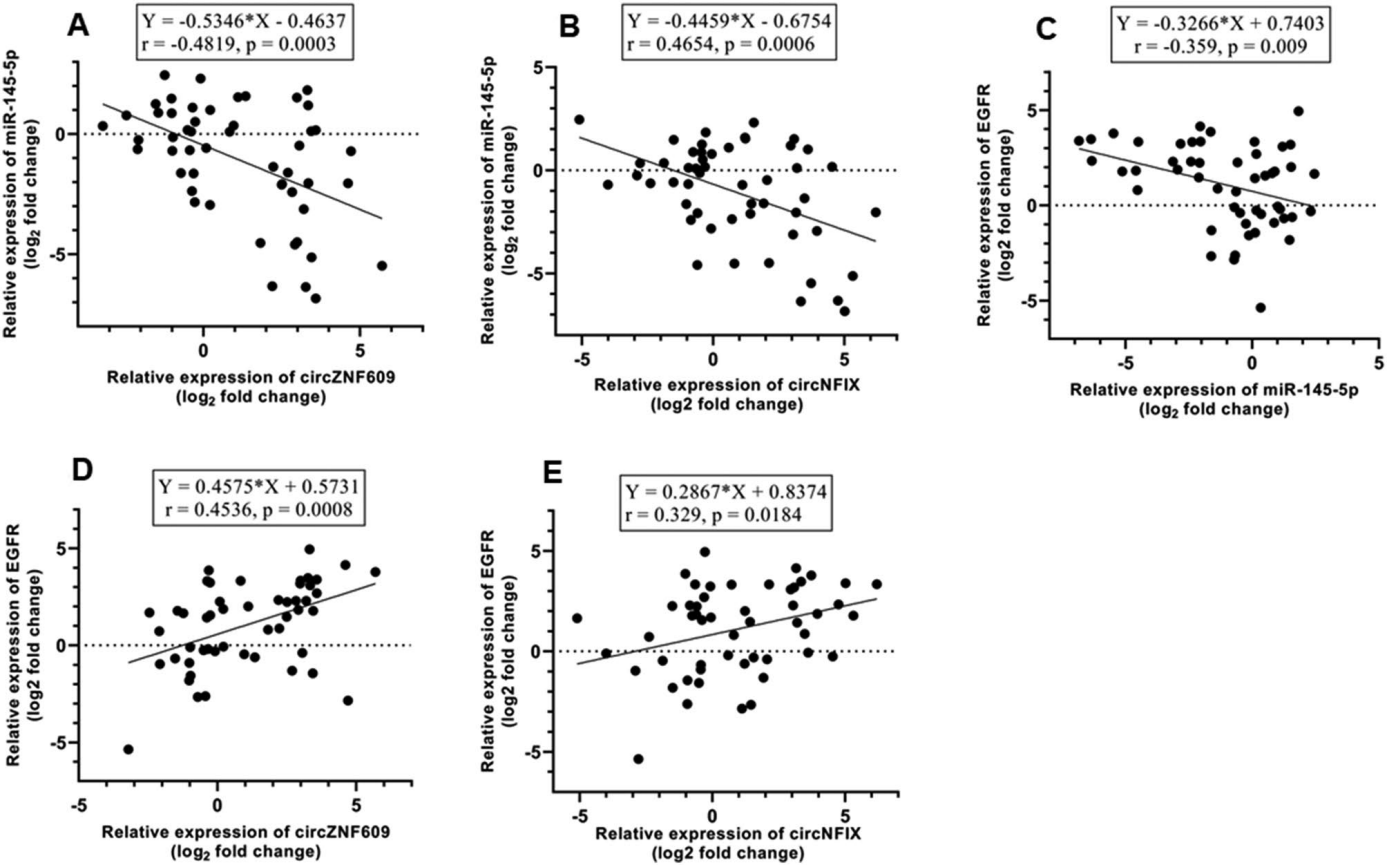
The correlation between expression levels of (**A**) *circZNF609* and *miR-145-5p*, (**B**) *circNFIX* and *miR-145-5p* (**C**) *miR-145-5p* and *EGFR*, (**D**) *circZNF609* and *EGFR*, and (**E**) *circNFIX* and *EGFR* genes in GBM tissues compared with normal brain samples.

Pearson’s correlation analysis showed the correlation between the expression levels of *cZNF609*, *cNFIX*, *miR-145-5p*, and *EGFR*. Results demonstrated a negative significant correlation of *circZNF609* and *circNFIX* with *miR-145-5p* (r = − 0.481, p = 0.0003; and r = − 0.465, p = 0.0006) (Fig. 6A, B), and a strong inverse correlation was observed between the expression of *miR-145-5p* and *EGFR* (r = -0.359, p = 0.009) (Fig. 6C). Notably, a significant positive correlation was observed between *circZNF609* and *circNFIX* with *EGFR* mRNA levels in GBM patients (r = 0.4536, p = 0.0008; r = 0.329, p = 0.018) (Fig. 6D, E).

**Figure 7:**
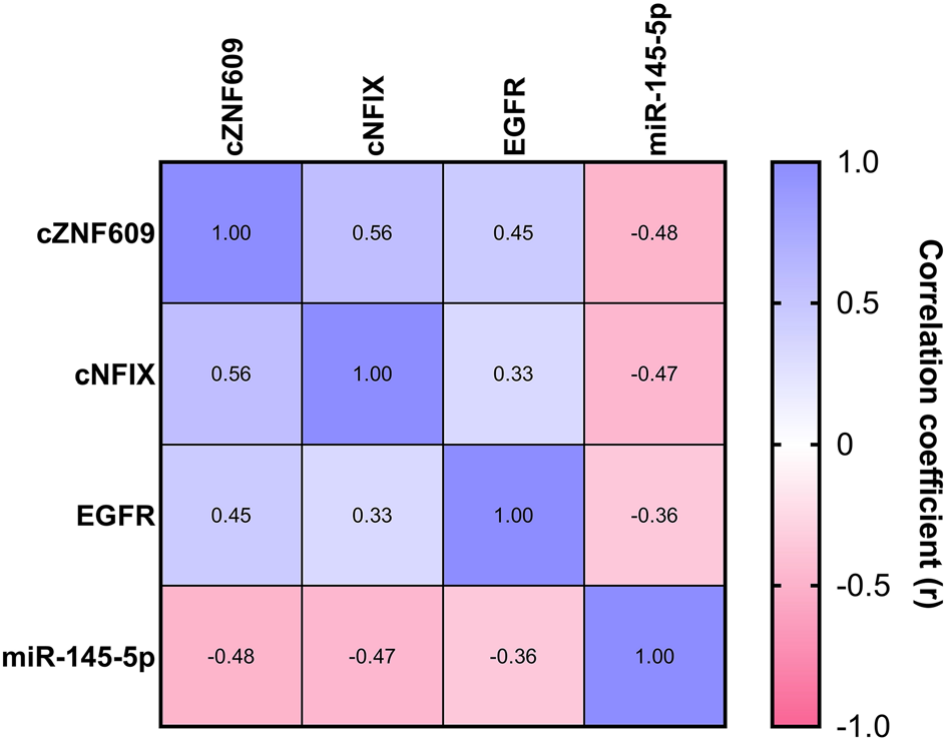
Grouped graph of expression correlation of *cZNF609*, *cNFIX*, *miR-145-5p* and *EGFR* in GBM and normal tissues as calculated by Pearson correlation.

The identified correlation pattern between *circZNF609*, *circNFIX*, *miR-145-5p* and *EGFR* is consistent with our hypothesis regarding the sponging effect of *cZNF609* (*circ0000615*) and *cNFIX* (*circ0049658*) on *miR-145-5p*. The expression correlation between *cZNF609*, *cNFIX*, *miR-145-5p* and *EGFR* in both tumor and normal tissues is represented by a grouped graph (Fig. 7).

### Receiver operating characteristic (ROC) curve analysis

**Figure 8:**
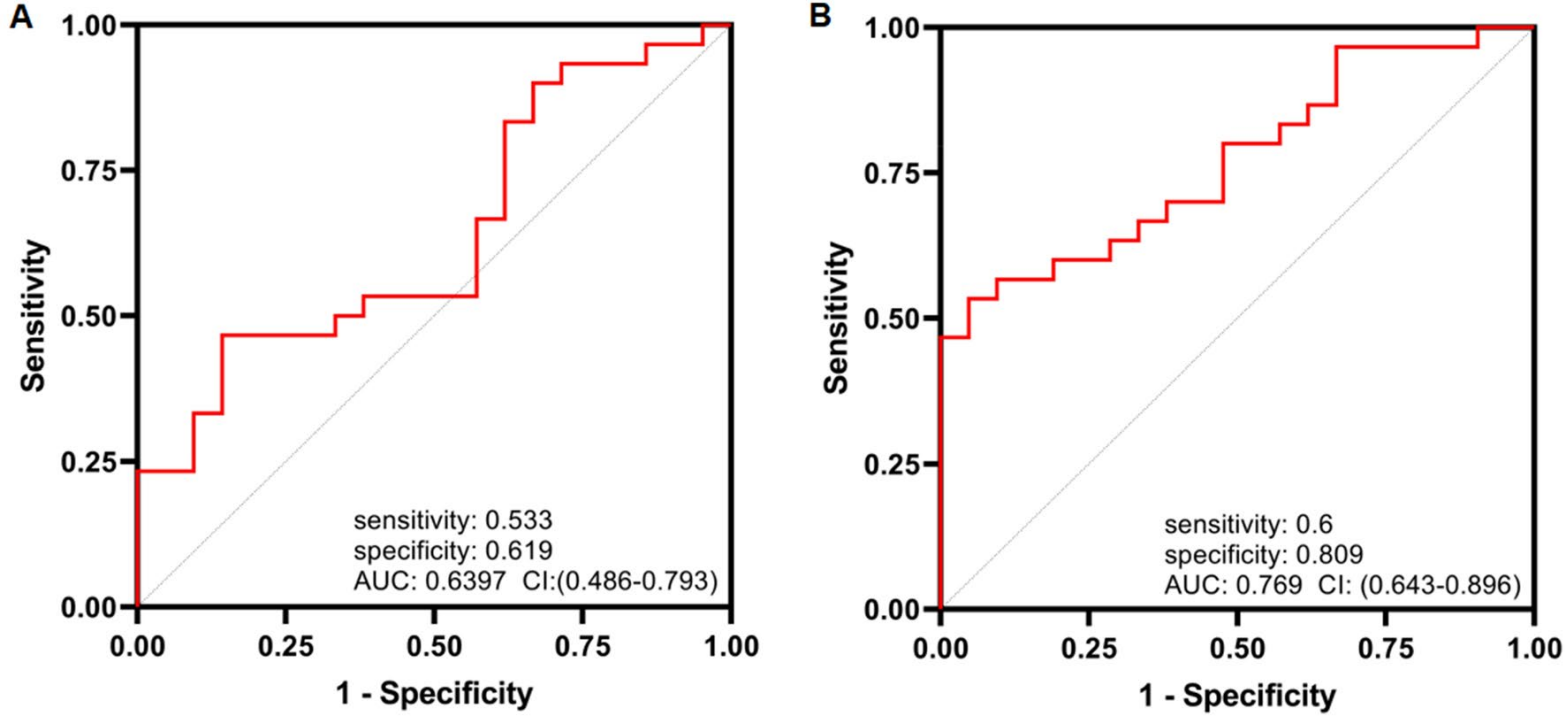
ROC curve analysis of (**A**) *circNFIX* and (**B**) *circZNF609* expression for discrimination of GBM tumors from normal tissues.

The possible clinical utility of *cNFIX* and *cZNF609* as a distinct GBM biomarker was evaluated by ROC curve analysis (Fig. 8A, B). The ROC curve analysis for *cZNF609* indicates that this biomarker was well able to discriminate between tumoral and non-tumoral tissues due to its high level of AUC, sensitivity, and specificity (p = 0.001) (Fig. 8B).

### Association between expression of *circZNF609*, *circNFIX*, *miR-145-5p* and *EGFR* and clinicopathological parameters

We assessed the association of *circZNF609*, *circNFIX*, *miR-145-5p* and *EGFR* expression levels in tumoral vs normal brain tissues with clinicopathological parameters. There was no significant difference in age, gender and tumor size among the groups (p > 0.05).

## Discussion

Glioblastoma is the most aggressive diffuse glioma of astrocytic lineage and is considered a grade IV glioma based on 2021 WHO classification of CNS tumors^22^. The current standard treatment for glioblastoma involves surgery, chemotherapy and, radiotherapy^23^. However, as the average overall survival of patients is low, new diagnostic and treatment approaches are needed. Due to the rapid progression, even with aggressive multimodal therapy, glioblastoma remains almost incurable^24^. It is known that one of the most important cell signaling pathways involved in the development of glioblastoma is epidermal growth factor receptor. The EGFR signaling cascade activates several intracellular signaling pathways, including the mitogen-activated protein kinase (MAPK), phosphoinositide 3-kinase (PI3K), signal transducer and activator of transcription 3 (STAT3) pathways, and thus can cause proliferation and tumor growth, angiogenesis, and alteration of the tumor microenvironment^25^. Existing evidence has demonstrated that *EGFR* overexpressed in ~ 60% of primary GBM patients. Therefore, a better understanding of the EGFR signaling network and its interactions with other pathways is essential to elucidate the mechanisms of resistance and to develop better therapeutic agents^24,25^. Today, cancer treatment has moved towards pathway-based biomarkers therapies^26^. The research has provided new insight to explore the molecular mechanisms involved in glioblastoma^3^. Many studies revealed that noncoding RNAs such as circRNAs, lncRNAs and microRNAs could regulate many important pathological processes including initiation, progression, metastasis, and drug resistance. Several lines of evidence indicate the role of circular RNA molecules in different types of cancers, including glioma^27^. However, the molecular mechanism of circRNAs remains largely unknown.

circRNAs are endogenous single-stranded RNAs with covalently closed-loop structures and are highly stable, conserved, and abundantly expressed in various tissues^28^. *Circ-ZNF609* (circBase ID: *hsa_circ_0000615*), with a full length of 874 bp, located at chr15: 64791491–64792365, is derived from the circulation of the second exon of *ZNF609*^29^. *Circ-NFIX* (*hsa_circ_0049658*) located in chr 19 (13183860-13192669), backspliced by exons 3-8 and 695 bp in length^30^. These circRNAs have highly conserved and fundamental biological functions among different species, suggesting that they play a critical role in cellular processes or organismal development^31,32^. *Circ-ZNF609* and *circ-NFIX* have been identified as circular RNAs expressed in neural tissue and may therefore play an important role in neural functions or processes^28^. Recently, several studies have reported dysregulation of *cZNF609* and *cNFIX* in various tumors and diseases^7,18,30,33,34^. In breast cancer, *circZNF609* plays an oncogenic role by elevating *p70S6K1* expression via sponging *miR-145-5p*^35^. According to the study by Pan et al., *circNFIX* attenuated pressure overload-induced cardiac hypertrophy by regulating *miR-145-5p*/*ATF3* axis^36^. Thus, *MiR-145-5p* might be sponged by *cZNF609* and *cNFIX*, and its expression was regulated by these circRNAs. Previous studies have shown that the expression of *miR-145-5p* is decreased in GBM patients, suggesting it can be a promising diagnostic and prognostic biomarker for this condition^37^. Another study reported that *circZNF609* promotes glioma cell migration and proliferation via the *miR-134-5p*/*BTG-2* axis^38^. In laryngeal squamous cell carcinoma (LSCC), elevated level of *circZNF609* has been associated with poor patient survival. Moreover, the functional investigation indicated that *circZNF609* regulated LSCC cell proliferation and invasion by sponging *miR-342-3p* and upregulating *EGFR*^8^. Xiao et al.^20^ demonstrated that *circNFIX* regulates the HCC progression and glutaminolysis by targeting *miR-3064-5p*/*HMGA2* axis. Xu et al., discovered through bioinformatics and experimental analysis that *circNFIX* promotes glioma progression by regulating *miR-34a-5p* via Notch signaling pathway. Several miRNAs, including *miR-145-5p*, were also reported to have binding sites with *circNFIX* in this study^7^. Therefore, aberrant expression of these circRNAs plays an important role in tumor proliferation, cell cycle regulation, invasion, and metastasis by modulating complementary miRNAs or target mRNAs linked to cancer-related signaling pathways^39–41^. These findings suggest that *circZNF609* and *circNFIX* may act as predictive biomarkers for cancer prognosis and as promising targets for cancer therapy. However, the specific role of *circ-ZNF609* in glioblastoma remains largely unexplored.

In this study, we first predicted the *circZNF609*, *circNFIX*/*miR-145-5p*/*EGFR* axis by extracting data from the literature and bioinformatics analysis using databases and web tools. Then, we evaluated the expression of each gene of this axis in 30 glioblastoma tissues and 21 normal brain tissues.

The results demonstrated that *circZNF609* expression was significantly up-regulated in tumor tissues (p = 0.001). CircNFIX gene expression was not significant in tumor versus normal samples (p = 0.06). In comparison, a study by Ding et al., performed on 65 glioma samples and 15 normal samples, showed that the expression level of *circNFIX* was significantly upregulated in glioma tissues compared to the normal control group, and there was a significant difference between the low and high grade groups. Furthermore, knockdown of *circNFIX* suppressed glioma progression in vitro and in vivo, possibly by regulating the miR-378e/RPN2 axis as a ceRNA^42^. This finding supports the previous studies indicating an oncogenic mechanism for *cZNF609* and *cNFIX* in different types of tumors^6,18,20,30^. Also, *miR-145-5p* and *EGFR* expression levels in glioblastoma tumors compared with normal brain tissues were decreased and increased, respectively (p = 0.0065, p = 0.002), while there was no statistically significant difference between expression levels of *circZNF609*, *circNFIX*, *miR-145-5p*, and *EGFR* genes with clinicopathological characteristics of patients, including age, gender and tumor size (p > 0.05). To use the relation of expression of these genes in clinic, further studies with more samples of different types of glioma are necessary. According to the ROC curve analysis, we found that *circZNF609* transcript levels had more than 70% specificity in this regard, indicating that this circRNA can serve as good predictive biomarker to distinguish malignant from non-malignant tissues. Therefore, in samples suspicious for this disease, assessing the expression level of *cZNF609* can be useful in evaluating the malignancy of the tissue. In contrast, the AUC of *cNFIX* was 0.636 with 53.3% sensitivity and 61.9% specificity in GBM patients (p = 0.1038) (Fig. 8A). Thus, it is not a good biomarker to discriminate tumor from normal tissues.

Our findings showed that the expression of *cZNF609* and *cNFIX* was significantly negatively correlated with the expression of *miR-145-5p* (r = − 0.481, p = 0.0003 and r = − 0.465, p = 0.0006, respectively). Additionally, *cZNF609* and *cNFIX* was highly positively correlated with *EGFR* expression (r = 0.453, p = 0.0008; r = 0.329, p = 0.018).

We also found an inverse correlation between *EGFR* and *miR-145-5p* expression levels (r = − 0.359 and p = 0.009). By analyzing *miR-145* expression levels in GBM patients in this present study, 16 out of 30 patients showed ≥ two-fold reduction of miR-145 expression, which was associated with upregulation of the *EGFR* expression.

Based on bioinformatics analysis, *EGFR* was identified as a putative target of *miR-145-5p* (Fig. 2). Three binding sites for this microRNA were predicted in the *EGFR* gene using miRTarBase (Fig. 3C). In addition, there is strong evidence of interaction between the *EGFR* and *miR-145* in glioma. Lu et al. showed that restoration of *miR-145* in glioma cells significantly reduced in vitro proliferation, migration and invasion. Overexpression of *miR-145* also reduced the expression of *ADAM17* and *EGFR*^14^. Based on these findings, we hypothesize that *miR-145* may modulate *EGFR* expression. However, we speculate that there may be other target genes that competitively interact with *miR-145*. These need further investigation.

The present study is limited by the relatively small number of patients. Further studies with a larger group of patients and an approximately equal number of male and female participants are needed to determine reliable prognostic value.

## Conclusion

Our study demonstrated that *circZNF609* and *circNFIX* may play a role in the pathogenesis of glioblastoma through the *miR-145*/*EGFR* axis, which requires functional studies. Further in vitro and in vivo model studies are needed to better understand the mechanism of this axis. Identifying molecular dysregulations in the EGFR signaling pathway may pave the way for potential therapeutic interventions and improve medical diagnosis.

### Supplementary Information

Supplementary Table 1.

## Data Availability

The datasets used and/or analyzed during the current study are available from the corresponding author on reasonable request.
